# Successful Management of a Gastric Sleeve Leak with an Endoscopic Stent

**DOI:** 10.1155/2012/205979

**Published:** 2012-05-17

**Authors:** Albin Abraham, Kaleem Rizvon, Jaspreet Singh, Ghulam Siddiqui, Apsara Prasad, Sadat Rashid, Magdalene Vardaros, Vikas Garg, Krishnaiyer Subramani, Paul Mustacchia

**Affiliations:** ^1^Department of Internal Medicine, Nassau University Medical Center, East Meadow, NY 11554, USA; ^2^Department of Gastroenterology, Nassau University Medical Center, East Meadow, NY 11554, USA

## Abstract

Laparoscopic sleeve gastrectomy has been a recently developed technique for treating morbid obesity. Gagner and Patterson performed the first laparoscopic sleeve gastrectomy as part of a duodenal switch procedure at Mount Sinai Hospital in New York in 1999. Since then many surgeons and institutions have adopted this technique. One of the most dreaded complications of sleeve gastrectomy is a leak along the staple line. We present the case of a 23-year-old female with gastric sleeve leak managed successfully with a fully covered wall flex stent. Our aim is to examine the incidence, causes, classification, and presentation of gastric sleeve leaks and to evaluate the use of endoscopic stents in its management.

## 1. Introduction

Laparoscopic sleeve gastrectomy has become a standard procedure for the surgical treatment of patients with different degrees of obesity. Clinical advantages include good weight loss; no rerouting of intestine which eliminates the risk of late bowel obstruction from internal herniation, and unlike the gastric band since there is no foreign body present, the risk of slippage and erosion is eliminated [1]. However this procedure causes three important adverse effects: staple line bleeding, strictures, usually at the middle or distal portion of the residual stomach, and stapler line leaks which causes the greatest morbidity. The treatment for leaks after sleeve gastrectomy varies and depends upon the extent of disruption, the extent of abdominal contamination, and the site of leak (proximal versus distal). Here we present a patient with a late gastric sleeve leak successfully managed with the placement of a fully covered wall flex stent.

## 2. Case Presentation

**Figure 1:**
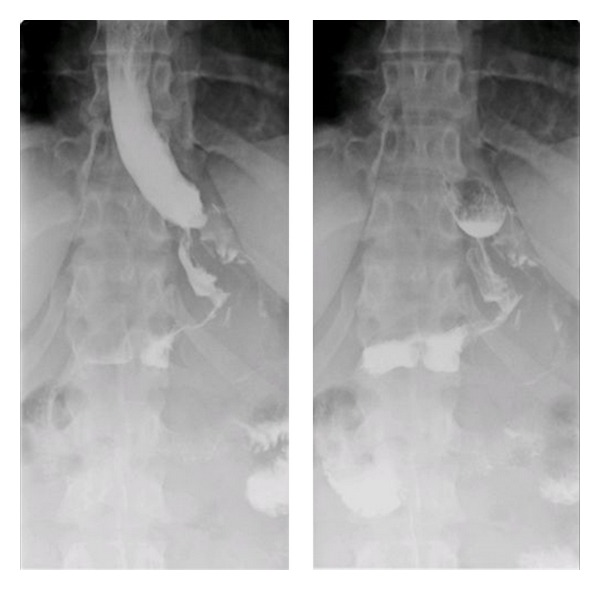
Upper GI series revealing gastric leak.

**Figure 2:**
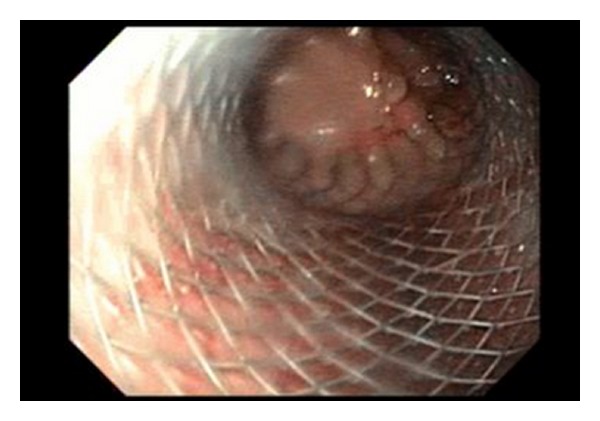
Gastric sleeve stent.

**Figure 3:**
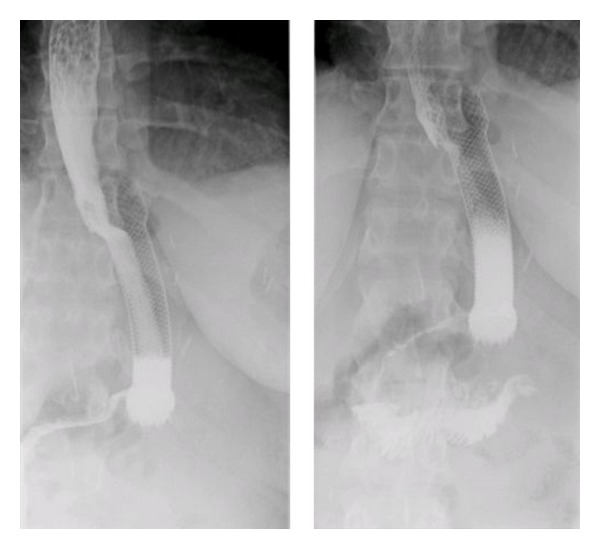
Upper GI series after stent placement.

**Figure 4:**
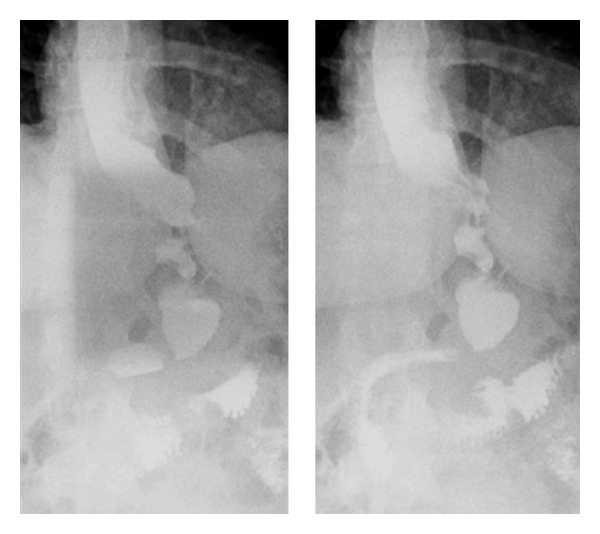
Upper GI series after stent removal.

A 23-year-old woman weighing 301 pounds and with a body mass index of 50.1 kg/m² presented to the bariatric surgery clinic for weight-loss surgery. She underwent a laparoscopic sleeve gastrectomy with no apparent postoperative complications. However, two weeks later, she presented with complaints of nausea, vomiting, epigastric pain and fever. She denied any hematemesis, changes in bowel movements, recent travel, or sick contacts. On examination she was febrile but had no peritoneal signs. CT abdomen revealed a 5.4 × 3 cm fluid collection abuting the gastric body. Upper GI series revealed a leak near the GE junction (Figure 1). Intravenous antibiotics and total parenteral nutrition were initiated. An upper endoscopy was done to assess the site and the size of the leak following which an 18 mm × 125 mm fully covered wall flex stent was placed (Figure 2). Gastrografin study during the procedure revealed no leak (Figure 3). The patient was discharged home on total parenteral nutrition. Followup demonstrated clinical improvement, and repeat CT abdomen showed improvement in the fluid collection. The patient was started on a clear liquid diet 2 weeks after the procedure, to which protein shakes were later added. Patient was able to tolerate pureed food without any complaints at the end of the second month. Gastric sleeve stent was removed after 6 weeks. Follow-up upper GI series showed no gastric leak (Figure 4). Proton pump inhibitor therapy was discontinued after 8 weeks. One year after the procedure the patient has lost 103 pounds and has been doing well.

## 3. Discussion

Laparoscopic sleeve gastrectomy is a new surgical procedure to tackle morbid obesity that has been gaining enthusiasm among both patients and surgeons. It restricts the stomachs size thereby inducing satiety and resects the fundal ghrelin—producing cells to decrease appetite [2]. Though this procedure may seem to involve less risk than gastric bypass and biliopancreatic deviation, its complications can be more challenging and increase morbidity and mortality. The most important complications are bleeding of the staple line in nearly 2%, stricture of the midportion of the tubular stomach in 1%, and gastric leaks with incidence varying from 0.7 to 20% [3]. The differences in incidence of gastric leaks can be explained on the basis of the expertise and practices of the respective endoscopist and the center at which the procedure is performed. Incidence rates tend to be higher at postgraduate teaching centers. Some centers also routinely perform radiologic contrast studies in every patient on the third day after the procedure with barium sulfate that has a much greater possibility of detecting small subclinical leaks which are not seen with a liquid contrast.

Csendes et al. have proposed a classification of leaks based on time of appearance after surgery, clinical severity, and location of leaks [4]. Leaks have been classified based on the period they appear as *early*, between first and fourth day post op, *intermediate*, between the fifth and ninth day after surgery, and *late,* appearing after day ten. *Type I* or subclinical are those that appear as a localized leak, without spillage, with few clinical manifestations and are easy to treat medically. *Type II* are those with dissemination into the abdominal or even pleural cavity. Most leaks appear in the proximal third of the stomach, close to the gastroesophageal junction. Burgos et al. reported 85.7% of leaks in the proximal third and only 14.3% in the distal third [5].

Most authors suggest that the major risk factor for gastric leaks is not staple line dehiscence but is due to ischemia in the gastric wall next to the staple line. Classic ischemic fistulas tend to appear between 5 and 6 days after surgery, when the wall-healing process is between the inflammation and fibrotic phase. Mechanical fistulas are usually discovered earlier, within the first 2 days after surgery [6].

Clinical picture of patients with gastric leak varies from the completely asymptomatic patient to sepsis, multiorgan failure, and death. The most common signs and symptoms were fever (81%), epigastric pain (69%), tachycardia (44%), and leukocytosis (75%) [7]. A methylene blue test, gastrografin study, and computerized axial tomography can aid in diagnosis. When an endoscopy is done to evaluate the site and size of the leak, carbon dioxide insufflation may be safer given its more rapid absorption.

The treatment of leaks after sleeve gastrectomy should be tailored to the clinical state of the patient. Those presenting with hemodynamic instability or with signs of sepsis require prompt surgical reintervention. The time of appearance and diagnosis of the leak is also crucial. Early leaks usually need a prompt surgical approach. On the contrary intermediate and late leaks are managed in majority of cases through medical treatment.

Medical management includes but is not limited to placing a drain if necessary, parenteral or enteral nutrition, high-dose proton pump inhibitors, broad spectrum antibiotics, use of biological glues (such as Seamguard, Tissucol, and fibrin sealant), and use of flexible coated stents. The use of stents in the management of gastric sleeve leaks have been gaining popularity with a few reports published on the outcome of the same. Nguyen et al. [1] reported successful deployment of a covered stent in a series of three patients with no complications relating to the stent placement. Serra et al. [2] reported the use of coated self-expanding stents for management of leaks after sleeve gastrectomy in six patients with control of leaks in 83%. Tan et al. [3] reported a 50% success rate for closure of gastric sleeve leaks with four patients requiring premature removal of the stent due to migration, hematemesis, or obstruction from kinking at the proximal aspect of the stent. Casella et al. [8] reported complete healing occurring in all three patients treated with an endoscopic stent for treatment of a leak after sleeve gastrectomy. Inbar et al. [9] reported five patients with esophago/gastric leaks or postoperative fistulas, in which self-expanding metal stents were successfully inserted and either retrieved or excreted following full recovery of all patients. Several considerations should be made when a stent is used for management of a gastric leak, after sleeve gastrectomy. An endoscopy should first be performed to evaluate the site of the leak, the size of the leak and viability of the conduit. Gastric leaks at the proximal and midaspect of the gastric sleeve are amenable to endoscopic treatment with a stent. A leak at the distal staple line, near the gastric antrum, would not be amenable to endoscopic stenting as the stent would be too small and would not provide appropriate sealing of the defect. The gastric sleeve diameter should also be evaluated at the time of endoscopy to aid in selection of the endoscopic stent size. A larger-sized stent would be less predisposed to migration. Another strategy to minimize stent migration would be to use a longer stent whereby the distal aspect of the stent is rested along the wall of the gastric antrum which precludes the stent from migration. Secondly any abdominal collection should be drained wither by laparoscopic or percutaneous means in combination with nothing per oral and nutritional support with either total parenteral nutrition or jejunostomy feeding [1].

Nausea, vomiting, drooling, early satiety, and retrosternal discomfort are the most common symptoms after stent placement and tend to disappear within the initial days. Stent migration is one of the main concerns in a third of the patients. When this occurs, the stent must be replaced, removed, relocated, or passed per rectum [2]. Most authors recommend a period of 6 to 8 weeks as the optimal time to withdraw the stent. This procedure can be challenging as adhesions due to the stent are frequent and therefore mucosal tears and bleeding can appear following the procedure [10].

While it is possible that our patient may have improved with solely conservative management, the use of a stent led to a more predictable outcome and a speedier recovery. However, given the complexities in management of gastric leaks, every effort must be made to prevent its occurrence. General surgical principles such as careful selection of patients, the experience of the surgeon, gentle handling of tissues, appropriate surgical techniques including careful management of electrocautery, and vessel sealing are essential. Some centers routinely perform the methylene blue test at the end of the surgery. However, a negative test only demonstrates that the surgical technique was appropriate at that time [7]. Therefore close clinical observations and early detection of gastric leaks are cornerstones of its appropriate management.
